# The *Rx for Change *database: a first-in-class tool for optimal prescribing and medicines use

**DOI:** 10.1186/1748-5908-5-89

**Published:** 2010-11-18

**Authors:** Michelle C Weir, Rebecca Ryan, Alain Mayhew, Julia Worswick, Nancy Santesso, Dianne Lowe, Bill Leslie, Adrienne Stevens, Sophie Hill, Jeremy M Grimshaw

**Affiliations:** 1 Institute of Population Health, University of Ottawa, 1 Stewart Street, Ottawa, ON, K1N 6N5, Canada; 2 Centre for Health Communication and Participation, Australian Institute for Primary Care and Ageing, La Trobe University, Melbourne, VIC, 3086, Australia; 3 Department of Clinical Epidemiology and Biostatistics, McMaster University, 1200 Main Street West, Hamilton, ON, L8N 3Z5, Canada; 4 Canadian Agency for Drugs and Technologies in Health, 600-865 Carling Avenue, Ottawa, ON, K1 S 5S8, Canada; 5 Clinical Epidemiology Program, Ottawa Health Research Institute, 1053 Carling Avenue, Administration Building, Room 2-017, Ottawa ON, K1Y 4E9, Canada; 6 Department of Medicine, University of Ottawa, Ottawa, ON, Canada

## Abstract

**Background:**

Globally, suboptimal prescribing practices and medication errors are common. Guidance to health professionals and consumers alone is not sufficient to optimise behaviours, therefore strategies to promote evidence-based decision making and practice, such as decision support tools or reminders, are important. The literature in this area is growing, but is of variable quality and dispersed across sources, which makes it difficult to identify, access, and assess. To overcome these problems, by synthesizing and evaluating the data from systematic reviews, we have developed *Rx for Change *to provide a comprehensive, online database of the evidence for strategies to improve drug prescribing and use.

**Methods:**

We use reliable and valid methods to search and screen the literature, and to appraise and analyse the evidence from relevant systematic reviews. We then present the findings in an online format which allows users to easily access pertinent information related to prescribing and medicines use. The database is a result of the collaboration between the Canadian Agency for Drugs and Technologies in Health (CADTH) and two Cochrane review groups.

**Results:**

To capture the body of evidence on interventions to improve prescribing and medicines use, we conduct comprehensive and regular searches in multiple databases, and hand-searches of relevant journals. We screen articles to identify relevant systematic reviews, and include them if they are of moderate or high methodological quality. Two researchers screen, assess quality, and extract data on demographic details, intervention characteristics, and outcome data. We report the results of our analysis of each systematic review using a standardised quantitative and qualitative format. *Rx for Change *currently contains over 200 summarised reviews, structured in a multi-level format. The reviews included in the database are diverse, covering various settings, conditions, or diseases and targeting a range of professional and consumer behaviors.

**Conclusions:**

*Rx for Change *is a novel database that synthesizes current research evidence about the effects of interventions to improve drug prescribing practices and medicines use.

## Background

The safe and effective use of medicines is an important aspect of quality healthcare. While there is an abundance of data on the clinical effectiveness and safety of various drugs, this does not ensure that the drugs are being appropriately prescribed or taken; in fact, suboptimal prescribing and medication errors are common across countries [1]. Research has indicated that guidance to health professionals and consumers alone does not reliably change behavior, and clinical practice is often based on personal beliefs rather than on scientific evidence [2]. In order to improve professional practice, approaches that have been shown to be effective should be used to promote optimal decision making and patient care [3]. To date, a large body of evidence evaluating the effectiveness of interventions to change clinical practice has been produced [4,5]. However, the volume of this literature, its wide dispersion, and its variable quality make it difficult for decision makers to access, assemble, and assess this evidence [6].

To address these problems, the Canadian Optimal Medication Prescribing and Utilization Service (COMPUS) program, within the Canadian Agency for Drugs and Technologies in Health (CADTH), and in collaboration with the Cochrane Effective Practice and Organisation of Care (EPOC) Group and the Cochrane Consumers and Communication Review Group (CC&CRG), have created and continue to update an online database of interventions to promote evidence-based prescribing and medicines use, called *Rx for Change *http://www.rxforchange.ca.

*Rx for Change *is a novel, publicly-accessible database that we initially developed and populated with review-level evidence, and made available online in April 2007. We update it regularly to reflect accumulating and changing evidence and provide decision makers with reliable, up-to-date, evidence-based information in the form of reader-friendly summaries. In the database, we present key findings from systematic reviews that evaluate the effects of interventions directed at professionals, consumers, and organizations in a systematic way. This paper describes the methods for developing and populating the *Rx for Change *database and highlights key content and the significance of the database for healthcare policy makers, researchers, professionals, and consumers.

## Methods

### Design and procedure

Our goal for the *Rx for Change *database is to provide an overall synthesis of the evidence from systematic reviews on the effectiveness of interventions for improving prescribing by healthcare professionals and medicines use by consumers. The methods that we used to populate the database parallels systematic review methodology. We use reliable and valid methods to search and screen the literature, and to appraise and analyse the evidence from relevant systematic reviews. We then present the findings in an online format which allows users to easily access pertinent information related to prescribing and medicines use.

### Contributors

In partnership with the Canadian Federal, Provincial, and Territorial Health Ministries, COMPUS identifies and promotes optimal drug therapy and encourages evidence-based information in decision making among healthcare providers and consumers. COMPUS hosts the *Rx for Change *database online in a publicly accessible format and has recruited additional funding for this project.

EPOC produces systematic reviews of interventions to improve healthcare delivery and healthcare systems, such as audit and feedback, distribution of educational materials, and decision-support tools using a well-established taxonomy of interventions and methods. In addition, EPOC has conducted overviews of existing Cochrane reviews as well as non-Cochrane systematic reviews to assess and synthesise the evidence in the area of professional behavior change [4,5]. For the *Rx for Change *database, EPOC maintains and updates the evidence from published systematic reviews on professional interventions that impact on the delivery of care, as well as organisational, financial, and regulatory interventions that influence prescribing behaviour. For the purpose of this paper, the methods used to identify and evaluate interventions targeting prescribing will be described.

The CC&CRG produces systematic reviews of interventions targeted at consumers (patients and their family members or carers) to promote consumer participation in healthcare. The CC&CRG has developed resources and tools to help organise and synthesise the evidence in relation to consumer communication and participation, and members of the group are currently undertaking an overview of systematic reviews of interventions directed at consumers to improve medicines use [7]. For the *Rx for Change *database, the CC&CRG is responsible for maintaining and updating the evidence on the effect of consumer-targeted interventions, such as providing consumers with information or education on medicines use, or promoting medicines self-management skills among consumers.

Using the combined expertise of the two Cochrane review groups, we developed the methods used for the synthesis and the presentation of the findings for this database. Human resources required in successfully maintaining *Rx for Change *equals four highly trained staff members shared between both Cochrane groups, in addition to supervision from senior research staff.

## Results

### Identifying systematic reviews: searching and screening

With the assistance of an information specialist, we conduct comprehensive regular searches of electronic databases, including MEDLINE, EMBASE, Database of Abstracts of Reviews of Effects (DARE), and the Cochrane Database of Systematic Reviews (CDSR). In addition, we systematically hand-search the CDSR and DARE databases as interventions targeting consumers' use of medicines are not well indexed.

**Figure 1:**
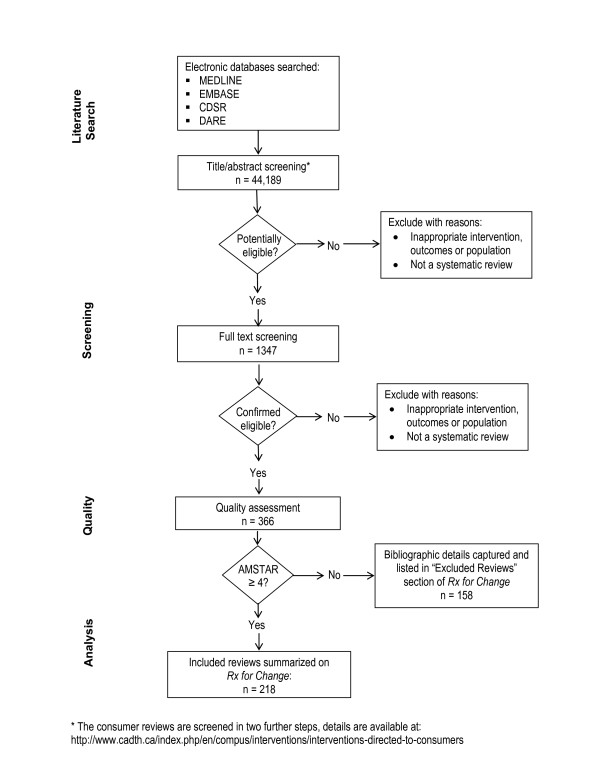
**Flow chart describing methods and procedures of *Rx for Change (as of April 2010)***.

Each year, we identify several thousand potentially eligible citations. Using explicit inclusion criteria, two researchers independently screen titles, abstracts and full text articles for relevance, and compare results. We resolve disagreements through discussion and, when necessary, through consultation with a senior team member (further details relating to selection criteria and methods are available online: http://www.cadth.ca/index.php/en/compus/optimal-ther-resources/interventions/methods). A flow diagram illustrating the methods and procedures for the database can be found in Figure 1.

### Quality assessment of systematic reviews

End users can be more confident in the results of systematic reviews that are of higher methodological quality. For this reason, two researchers critically appraise each review identified as eligible for inclusion in the database using the AMSTAR tool, a validated instrument for appraising systematic reviews [8]. AMSTAR is an 11-item checklist on which reviews score one point for each criterion met. Items assess methodological criteria such as the comprehensiveness of the search used and whether the quality of included studies was evaluated and accounted for. In consultation with AMSTAR developers, we created decision rules for each of the 11 items to facilitate an objective and consistent assessment across reviews.

Reviews are eligible to be summarised on the database if they achieve an AMSTAR score greater than 3. This decision was based on our experience that it is difficult to draw meaningful conclusions based on data from low-quality reviews. We make the bibliographic details and AMSTAR scores of these reviews available on the database under the heading 'Excluded Reviews.' To date, we have assigned approximately two-thirds of eligible reviews an AMSTAR score greater than 3, and have summarised these reviews on the database.

### Data extraction

When deciding what information should be abstracted from the individual reviews, we focused on information that is useful to decision makers. Two researchers independently extract data on demographic details, intervention characteristics, and outcomes from each review using a standardised data extraction form and a consensus process. This ensures a consistent summary format for each review and ensures the accuracy of the information.

### Analysis and synthesis

We analyse, summarise, and report separately the results of all relevant comparisons within each systematic review using quantitative and qualitative methods as appropriate. Because reviews vary greatly in the type and amount of study data reported, we often use vote counting for data synthesis to allow for consistent presentation of results. We report our analyses by vote counting as the number of studies that favor the intervention (based on direction of effect) out of the total number of studies for each comparison. We also include any additional review data, such as meta-analyses or effect sizes. We then compile the results from each comparison and present them in a 'Table of Results.' We use standardised decision rules and statements to descriptively report on the general and medicines-specific 'Results' and 'Conclusions' of each review. For example, we use the term 'generally effective' if two-thirds or more of the studies favor the intervention.

We organize the reviews of interventions directed to professionals and the delivery of care using the EPOC taxonomy of interventions. This taxonomy groups interventions into five broad categories: interventions targeting healthcare professionals, changing the organization of healthcare, financial interventions, regulatory interventions, and structural changes. Each category includes a number of specific interventions. Examples of interventions targeting professionals include reminders, educational meetings, and audit and feedback. Because the consumer literature on medicines use had not been previously well organised, the CC&CRG developed a taxonomy of consumer directed interventions [9,10]. Examples of interventions include the provision of information or education and behaviour change support. We provide definitions of each intervention on the database. For each intervention, we summarise the evidence from all of the relevant systematic reviews. We create each intervention summary based on our findings from high quality and key reviews, and this includes a statement of the overall effectiveness of the intervention and the findings as they relate to prescribing and medicines use. For those interventions where no reviews were identified, we include a comment in the database, informing the users that there is a lack of review evidence.

With each update of the database, we combine new evidence with existing evidence and intervention summaries are updated. We display flags that indicate which interventions have been recently updated with new evidence.

**Figure 2:**
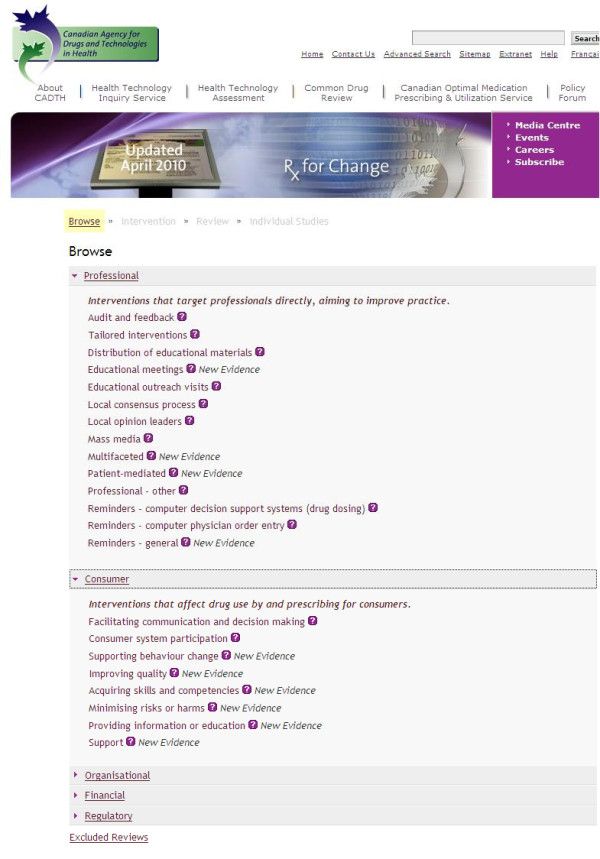
**Screenshot 1 of *Rx for Change *database**. Level 1: Identifies intervention categories (professional, consumer, organizational etc.) and specific interventions (audit and feedback, acquiring skills and competencies, *et al*.).

**Figure 3:**
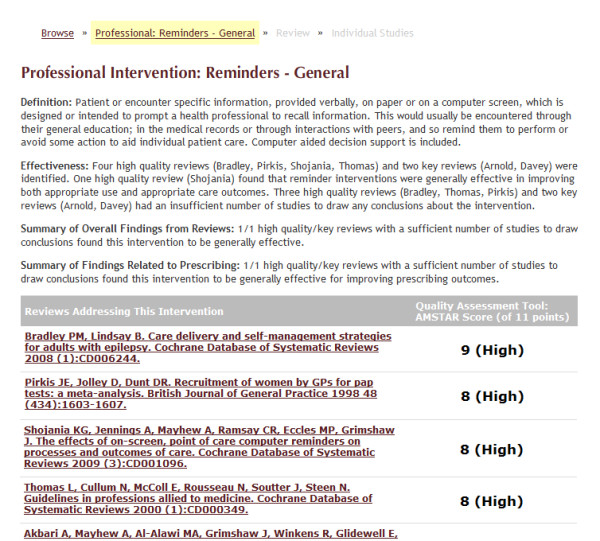
**Screenshot 2 of *Rx for Change *database**. Level 2: Provides evidence summaries within each intervention, with links to systematic review-level evidence summaries.

**Figure 4:**
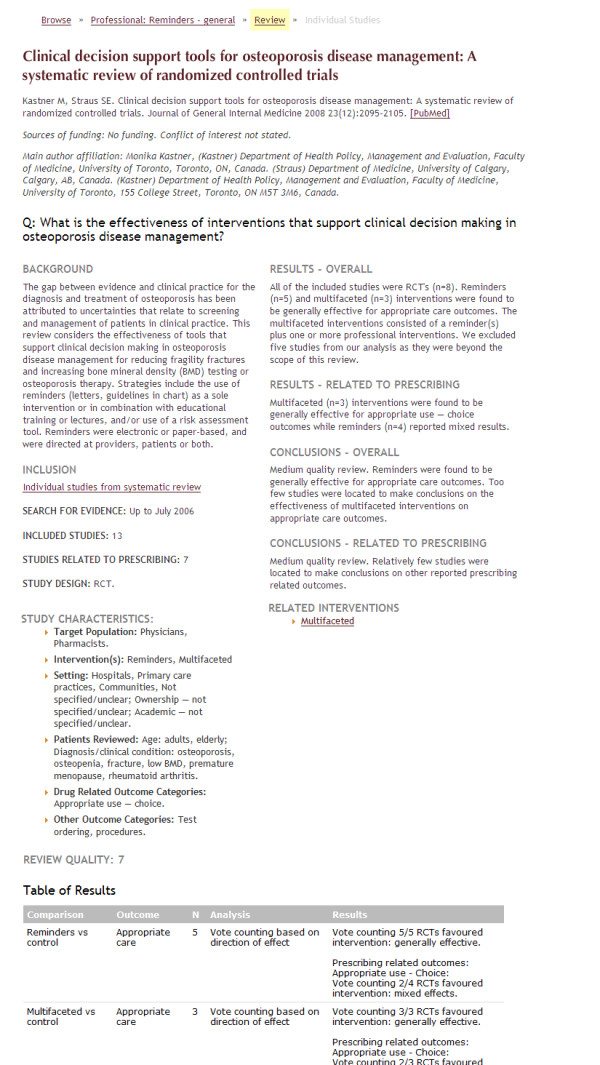
**Screenshot 3 of *Rx for Change *database**. Level 3: Provides synthesis of systematic review-level evidence.

**Figure 5:**
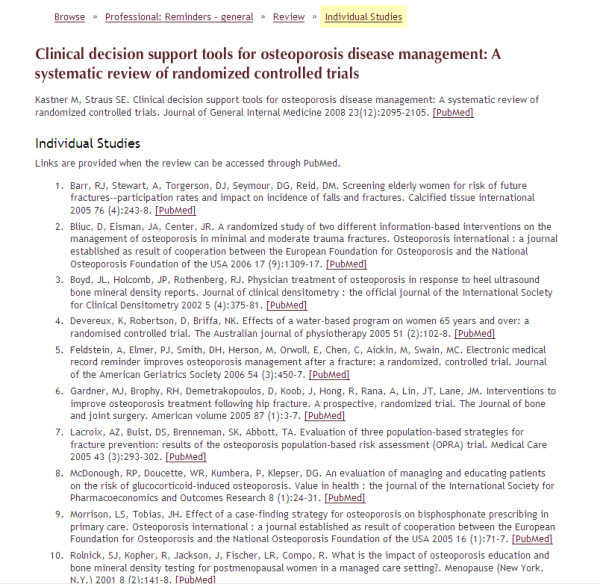
**Screenshot 4 of *Rx for Change *database**. Level 4: Provides a list of included studies within a systematic review, with links to PubMed.

We present the database in a multilevel approach. In the first level, we provide a list of interventions grouped into five categories: professional, consumer, organisational, financial, and regulatory (Figure 2). In the second level, we provide intervention summaries based on the findings from high quality and key systematic reviews (Figure 3). In the third level, we provide a summary of findings from the included studies in each systematic review (Figure 4). In the fourth level, we provide links to the studies included in each systematic review (Figure 5).

## Implementation

We launched the database in April 2007 and have since updated it three times (Table 1). We initially populated it with approximately 50 reviews, and it now contains over 200 summarised reviews (as of April 2010). The reviews that we have included in the database are diverse, spanning various settings, conditions, or diseases, and targeting a range of professionals, healthcare systems, and consumers. Details regarding the epidemiology and quality of reviews included in the *Rx for Change *database on professional behaviour change [11] and consumer-focused interventions [7] can be found elsewhere.

**Table 1:** Number of systematic reviews on *Rx for Change *at each update

Update	No. consumer reviews	No. professional reviews
April 2007	21	23

April 2009	33	82

October 2009	53	124

April 2010	63	155

## Discussion

*Rx for Change *is a well-designed database containing valuable information for researchers, healthcare providers, and policy makers. Since its inception, we have received positive feedback about the database from international users about its value, applicability, and quality. Within a year of its launch, it had accumulated more than 25,000 page views. With increasing awareness of the database and its ongoing updates, we anticipate that this interest will continue to grow. We will continue to disseminate key messages to local and international groups about which interventions are effective, and where gaps in the evidence exist. We will continue to explore methods to disseminate and translate key messages to end users, particularly as new evidence is found and added to the database.

The *Rx for Change *database has provided the opportunity for EPOC and CC&CRG to collaborate with organizations that have strong links with healthcare decision makers (*e.g*., CADTH, National Prescribing Service Australia). This collaboration promotes the use of research evidence and ensures that the data is available to the general public, healthcare professionals, and policy makers.

## Summary

We created the *Rx for Change *database to facilitate and improve the processes of accessing, searching, identifying, and using research to inform evidence-based prescribing and medicines use. It provides reliable, up-to-date, evidence for a wide range of users and is organised in an easy-to-browse format. We take the quality of the evidence into consideration to provide useable summaries that are relevant to decision-makers. This database is a first-in-class tool, and we will continue to promote it to ensure that it is utilized to its full potential.

## Competing interests

BL is currently employed by CADTH. MW, AM, JW, and AS have been or are currently employed by EPOC. JG is the Coordinating Editor of EPOC. RR, NS and DL are currently employed by CC&CRG. SH is the Coordinating Editor of CC&CRG.

## Authors' contributions

MW participated in the design, data collection, analysis, coordination of the study, and drafted the manuscript. RR, AM, JW, NS, DL, AS, and SH participated in the design, data collection, analysis, and coordination of the study and contributed to the manuscript. BL participated in the design of the study and provided feedback on the manuscript. JG conceived of the study, participated in its design and coordination and provided feedback on the manuscript. All authors read and approved the final manuscript.
